# Genetic Diversity Among Wild and Cultured Bighead Carp (*Hypophthalmichthys nobilis*) in the Middle Yangtze River by Microsatellite Markers

**DOI:** 10.3390/genes16050586

**Published:** 2025-05-16

**Authors:** Junru Wang, Qi Lei, Hanjun Jiang, Jun Liu, Xiaomu Yu, Xusheng Guo, Jingou Tong

**Affiliations:** 1 School of Fisheries, Xinyang Agriculture and Forestry University, Xinyang 464000, China; jrwang1021@163.com (J.W.); aqualeiq@163.com (Q.L.); 2021210003@xyafu.edu.cn (H.J.); liujunhnsd@126.com (J.L.); 2 Fishery Biological Engineering Technology Research Center of Henan Province, Xinyang 464000, China; 3 Institute of Hydrobiology, Innovation Academy of Seed Design, Chinese Academy of Sciences, Wuhan 430072, China; xmyu@ihb.ac.cn

**Keywords:** bighead carp, microsatellite markers, genetic diversity, wild, cultured

## Abstract

Background: Bighead carp (*Hypophthalmichthys nobilis*), a vital species in China’s freshwater ecosystems and aquaculture, has experienced significant population declines due to habitat degradation and intensive farming. Methods: In this study, eight polymorphic microsatellite markers were utilized to examine the genotypes and genetic diversity of 320 individuals of bighead carp populations located in the middle Yangtze River. This included four wild populations (ZX, DTH, SS, WH) and six cultured populations (HH, XZ, CH, QC, CD, HG). Results: Wild populations exhibited significantly higher genetic diversity (mean Na = 12.25 ± 0.63, Ho = 0.802 ± 0.063) than cultured groups (mean Na = 8.85 ± 1.21, He = 0.779 ± 0.032). Low differentiation (Fst < 0.05) among wild populations indicated high connectivity with low genetic structure, whereas cultured populations CH and HG showed moderate-to-high differentiation (Fst = 0.156–0.293). Bayesian analysis (K = 7) revealed a distinct clustering of wild populations, while cultured stocks exhibited admixed genetic ancestries. Bottleneck tests confirmed recent genetic bottlenecks in three cultured populations. Conclusions: Wild bighead carp populations retain a critical genetic diversity, serving as reservoirs for conservation, while intensive aquaculture practices threaten genetic integrity through allele loss and inbreeding.

## 1. Introduction

The investigation into the formation of and variation in gene frequencies and genotype frequencies is fundamental in population genetics. These frequencies elucidate the genetic composition of populations and enable predictions about alterations in genetic structures due to either natural or artificial selection [1]. Consequently, the sustainable development of fisheries and the conservation of aquatic ecology can be assessed through examining the genetic diversity of fish populations. Microsatellite markers (single sequence repeats), representing the second generation of molecular marker technology, present several advantages. When juxtaposed with traditional isozyme markers, Restriction Fragment Length Polymorphism (RFLP), Random Amplified Polymorphic DNA (RAPD), and Amplified Fragment Length Polymorphism (AFLP), microsatellite markers exhibit superior polymorphism, enhanced primer versatility, and high reproducibility [2]. Typically, these markers are not influenced by environmental factors, but it depends on where the DNA microsatellite is located. Moreover, these markers are not influenced by environmental or age factors, making them especially suitable for fish genetic diversity research [3]. Population genetics often compares the genetic diversity between wild and farmed populations, as exemplified by the research on Atlantic salmon by Skaala et al. [4] and the grass carp (*Ctenopharyngodon idella*) populations in the Yangtze River Basin [5].

Bighead carp (*H. nobilis*), a member of the order Carpiformes, family Carpidae, and genus *Hypophthalmichthys* [6], holds significant importance in China’s freshwater aquaculture sector. The cultivation of bighead carp provides various advantages such as a simple feeding regime, cost-effectiveness, and maintaining the balance of the aquatic ecosystem. The Yangtze River serves as the primary natural breeding ground and germplasm repository for this species [7]. However, human activities and water conservancy projects have contributed to a decline in the wild populations of bighead carp within the Yangtze River system [8], disrupting its genetic diversity. Currently, it is imperative to elucidate the status of bighead carp germplasm resources and genetic diversity in the Yangtze River system, given its significance for the sustainable development of the fishery industry. Hubei Province, where the middle Yangtze River flows, is currently the leading producer of bighead carp in China. Nevertheless, issues persist, such as the potential degradation of the germplasm due to the lack of modern scientific guidance and standardization in the artificial propagation process [9].

So far, there are few studies associated with genetic analyses comparing wild and farmed populations of bighead carp in the middle Yangtze River. In the present study, we used eight microsatellite markers to investigate and assess the differences between the genetic diversity of wild and cultured populations of bighead carp, which are useful for the sustainable development of fisheries in Yangtze River.

## 2. Materials and Methods

### 2.1. Fish Collection and DNA Extraction

**Figure 1:**
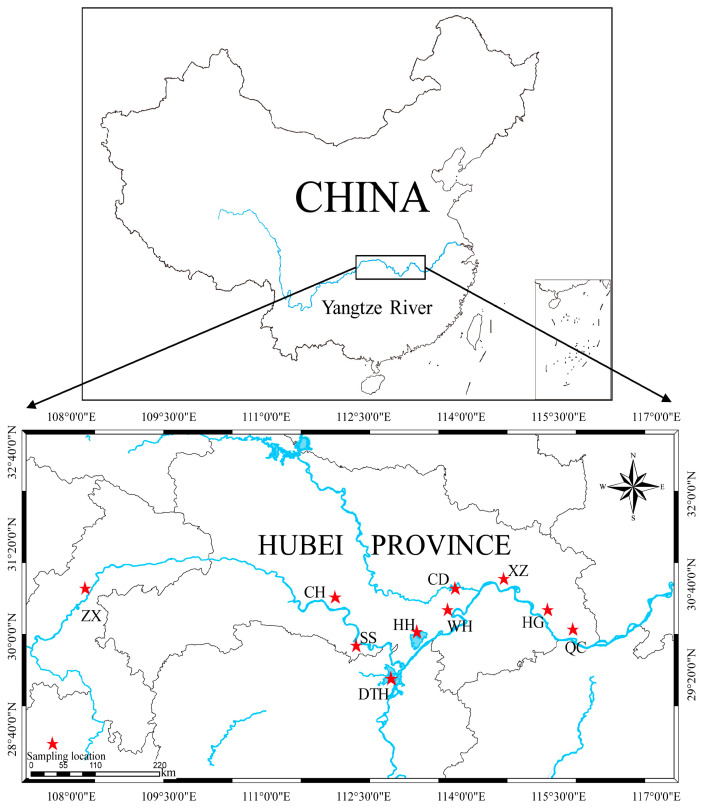
A map of ten populations of bighead carp in this study.

A total of 320 samples of bighead carp were involved in this study (detailed sampling information is provided in Table 1 and Figure 1). Three wild populations were sampled from Dongting Lake (DTH), Shishou (SS) in Jingzhou, and Wuhu Fish Farm (WH) in Xiantao. Additionally, six farmed populations, comprising adult fish purchased from markets and fry hatched in farms, were sampled from various locations: Honghu Lake (HH) and Changhu Lake (CH) in Jingzhou and Caidian (CD) and Xinzhou (XZ) in Wuhan, as well as Qichun (QC) in Huanggang and a farm location (HG). Zhongxian (ZX), a typical site selected for the first phase of ecological fisheries in the Three Gorges Reservoir Area, prioritizes both economic development and water’s ecological security. Given its unique geographical location, ZX offers significant advantages for the growth and restocking of bighead carp. Therefore, this study included bighead carp samples from ZX to facilitate a more comprehensive comparison of genetic diversity between farmed and wild bighead carp populations in the middle reaches of the Yangtze River.

Samples were collected from flipper or whole fish tissue from larvae, fixed in anhydrous ethanol and stored in a 4 °C refrigerator. Genomic DNA was extracted using the classic phenol–chloroform method. The DNA working solution was uniformly diluted to 50 ng/μL after determining the concentration of the DNA samples.

### 2.2. Microsatellite Marker Selection and PCR Amplification

The microsatellite markers used in this study were of bighead carp developed by the Institute of Aquatic Biology, Chinese Academy of Sciences [10]. The microsatellite primers and fluorescent primers used were provided by Shanghai Sangong Bioengineering Co. (Shanghai, China). The 5′ end of the upstream primer was modified with a FAM or HEX fluorescent group (Table 2). The total volume of the PCR reaction was 12.5 μL, and the reaction mixture contained the following components: 30–50 ng DNA template 1 μL, 0.25 μL Taq DNA polymerase, 1.25 μL 10 × PCR buffer, 0.4 μL dNTP (2.5 mmol/L), 0.4 μL forward and reverse primers mixture (2.5 μmol/L each), and 9.2 μL sterilized deionized water. The PCR amplification conditions were as follows: pre-denaturation at 94 °C for 5 min; 38 cycles of amplification, each cycle involving denaturation at 94 °C for 35 s, annealing at 48–60 °C for 35 s, and extension at 72 °C for 40 s; and final extension at 72 °C for 8 min.

### 2.3. Microsatellite Size Determination and Analysis

The PCR products were sequenced by Acorn Health Biotechnology Ltd. using a 3730 DNA sequencer (ABI, CA, USA), in which the internal standard used was Liz500 [11]. The sequencer was used to perform microsatellite typing to obtain the allele data of each individual at different loci, and the fragment size of the amplification products was read using GeneMarkerV2.2.0 software.

Genotypes were determined from the amplification product gene fragments at each locus. MS-TOOLS software was used to calculate diversity parameters such as observed heterozygosity (Ho), expected heterozygosity (He), number of alleles (Na) and calculated polymorphism information content (PIC).

Population genetic bottleneck effects were analyzed using Bottleneck 3.4 software, based on the infinite allele model (IAM), stepwise mutation model (SMM), and two-stage model (TPM), and heterozygosity was analyzed using the sign test and Wilcoxon signed-rank test to determine whether heterozygosity was significant. The sign test and Wilcoxon signed-rank test were used to analyze whether the heterozygosity was significant or not.

Nei’s genetic distances of the 10 populations and similarity coefficients were assessed by PopGene32 [12]. In this study, 10 bighead carp populations were clustered based on Nei’s genetic distance using the UPGMA method in MEGA 7.0 software [13]. To visualize the coordinates of similarities or differences between groups, we performed a Principal Coordinate Analysis (PCoA). The Fixation index (Fst) among the 10 populations was calculated using Arlequin 3.1 software [14], in addition to the Hardy–Weinberg equilibrium (HWE) test for each population. When performing multiple comparisons, the significance level of the probabilities required Bonferroni correlation [15].

Populations’ genetic structure was calculated using analysis of molecular variance (AMOVA). Bottleneck effect analysis was performed using Bottleneck 3.4 based on allele frequencies at each locus. The Bayesian approach hybrid model in Structure 2.2 was utilized to construct the population’s genetic structure map. The K values were set from 1 to 10, using a 10,000 burn-in length, while the Markov Chain Monte Carlo (MCMC) was set to 100,000 iterations, and each K was repeated 20 times. The K corresponding to the maximum value of ΔK was selected as the best K value, and the results were calculated online by Structure Harvester.

## 3. Results

### 3.1. Polymorphism Information of Microsatellite loci

A genetic information table for eight microsatellite markers of bighead is presented in Table 3. The Na varied from 10 (Arsd23) to 35 (Hysd293-1). The Ne was between 5.8 (Arsd241) and 14.7 (Hysd293-1). The Ho varied from 0.681 (Arsd241) to 0.887 (Hysd6). The He varied from 0.828 (Arsd241) to 0.934 (Hysd293-1). The PIC varied from 0.664 (Arsd241) to 0.852 (Hysd293-1). These values suggest that all eight loci are highly polymorphic, given that their PIC exceeds 0.5. The Fst varied from 0.057 (Hysd792-1) to 0.161 (Arsd241).

### 3.2. Genetic Diversity Within Populations

The genetic diversity data from the ten bighead populations (Table 4) revealed that the Na varied from 6.38 (HG) to 12.75 (DTH), while the Ne varied from 3.78 (HG) to 8.06 (DTH). The He in the cultured varied from 0.642 (CH) to 0.842 (CD), with an average of 0.779, while in the wild it ranged from 0.620 (CH) to 0.855 (HG), with an average of 0.869. The Ho in the cultured varied from 0.642 (CH) to 0.842 (CD), with an average of 0.794, while in the wild it ranged from 0.760 (SS) to 0.848 (WH), with an average of 0.814.

The PIC in the wild and cultured populations ranged from 0.613 to 0.853. The mean PIC value was 0.839 in the wild population and 0.740 in the cultured population. Notably, the levels of Na, Ne, and PIC were significantly higher in the wild population compared to the cultured population (*p* < 0.05).

### 3.3. Genetic Differentiation and Genetic Distance Analysis Among Populations

**Figure 2:**
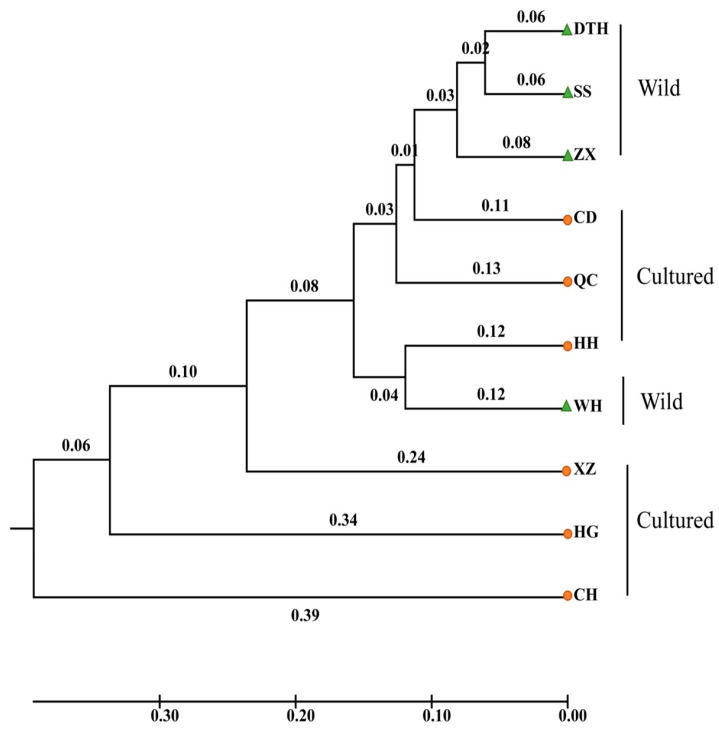
UPGMA trees of ten populations in bighead carp. Green triangles indicate wild populations; orange dots indicate farmed populations.

The genetic distances and Fst among the ten populations of bighead carp (Table 5) demonstrated that the genetic distances among these populations ranged from 0.122 (SS vs. DTH) to 0.827 (HG vs. CH), and the Fst varied between 0.003 (SS vs. DTH) and 0.293 (HG vs. CH). All Fsts reached the significance level (*p* < 0.05). The UPGMA phylogenetic tree (Figure 2) showed that the wild groups (ZX, DTH, and SS) formed one cluster, while the wild group WH and the cultured group (HH) formed another cluster. The cultured groups HG and CH established their own clusters. The HWE test revealed that populations HH, XZ, CH, CD, ZX, and HG deviated from Hardy–Weinberg equilibrium at the Hysd443-2 locus (Table 6).

**Figure 3:**
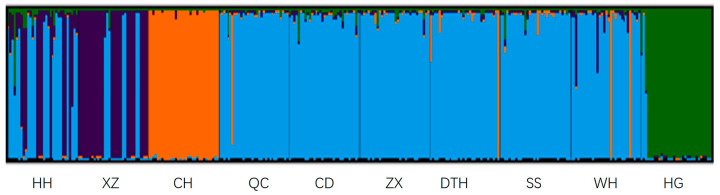
Bayesian clustering relationships of ten populations in bighead carp using the most likely K value (K = 4).

**Figure 4:**
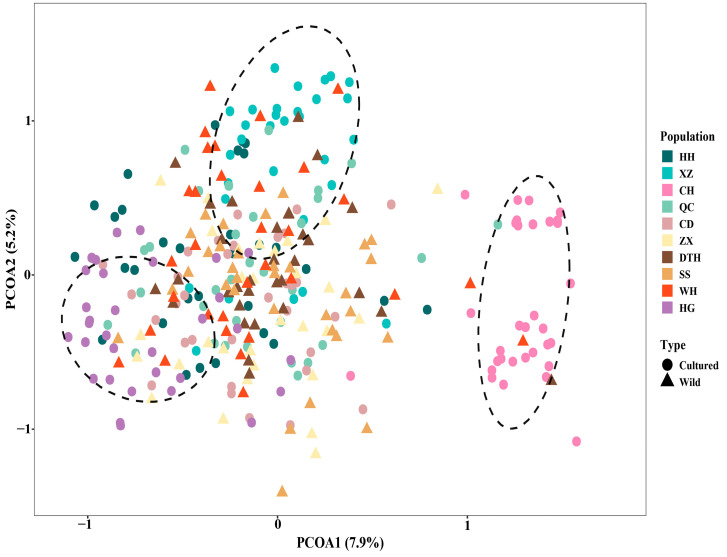
Principal Coordinate Analysis (PCoA) of 320 individuals from 10 bighead populations. Population abbreviations are shown in Table 1.

**Figure A1:**
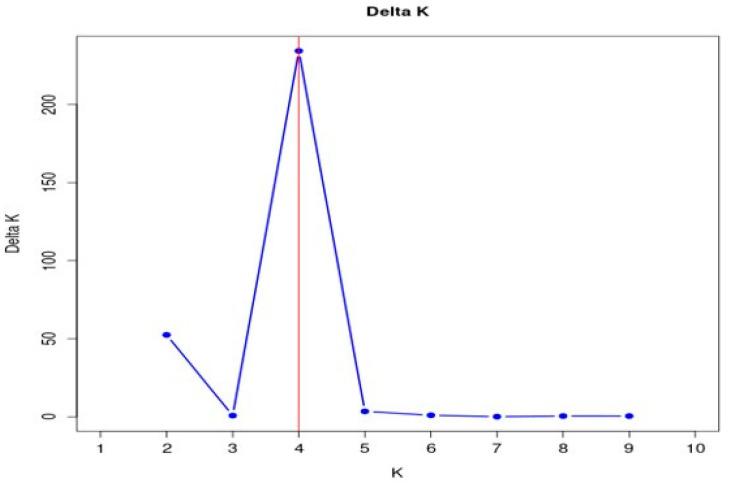
∆K values for K = 1–10 using Structure Harvester. K = 4 is shown as the optimal value.

Genetic bottleneck effect analysis (Table 7) demonstrated that all populations, with the exception of CH, WH, and HG, significantly deviated from mutation–drift equilibrium under the IAM model (*p* < 0.05). However, CD appeared to deviate from this equilibrium under the TPM model. AMOVA analysis showed a 1.58% genetic variation between wild populations and a 98.42% genetic variation within populations. The genetic variation among the cultured populations was 14.12%, and the genetic variation within the populations was 85.88%. For the mixed-model analysis, models with K-values ranging from 1 to 10 were selected. Using Structure Harvester, a K-value of 4 was identified as the most suitable fit (Figure A1). The Bayesian clustering relationship among the ten bighead carp populations is depicted in Figure 3. Both CH and HG showed unique taxa in the analysis, which is consistent with the differentiation results of the phylogenetic tree (Figure 3). The results of the PCoA analysis matched with the phylogenetic tree results. Wild groups (ZX, DTH, SS) were concentrated on PC1 and showed genetic similarity. Cultured groups (HH, QC, CD) were more widely dispersed (Figure 4). CH and HG were both shown to be unique taxa in the analysis, with significant genetic differentiation.

## 4. Discussion

### 4.1. Levels of Genetic Diversity of Microsatellite loci in Populations

Microsatellites, as commonly used molecular genetic markers for populations’ genetic analysis, are associated with polymorphisms derived from the number of core repeat sequence repetitions. The eight bighead carp microsatellite markers used in this study were all 4–5 base repeat sequences. PIC is an important reference standard for evaluating microsatellite loci, which are highly polymorphic when PIC > 0.5, moderately polymorphic when 0.25 < PIC < 0.5, and lowly polymorphic when PIC < 0.25 [16]. The PIC values of the microsatellite markers in this study ranged from 0.664 to 0.852, and all of them were highly polymorphic (Table 3). The observed heterozygosity and expected heterozygosity were both high (Ho > 0.68, He > 0.8); therefore, the eight loci used in this study were genetically diverse.

In addition, polynucleotide repeats in microsatellite markers, with a higher amplification stability than dinucleotide repeats, can more faithfully respond to the genetic nature of DNA sequences [17]. In this study, the number of alleles at microsatellite loci in the bighead population ranged from 10 to 35 (Table 3), indicating that all eight microsatellites were highly polymorphic seats. Therefore, the bighead carp satellite loci selected in this study are effective molecular genetic markers for exploring the genetic diversity of bighead carp populations.

### 4.2. Genetic Diversity Analysis of Bighead Carp Populations

The genetic diversity of a population is one of the most important bases for evaluating the status of a species’ germplasm resources. Maintaining the level of genetic diversity within a species is the ultimate goal of germplasm conservation and the basis for the sustainable utilization of germplasm resources [18]. The numbers of effective alleles, heterozygosity, and polymorphic information content of a population are all measures of populations’ genetic diversity, and higher values of these are indicative of the higher genetic richness of a population [19].

Ho and He are two important indicators of genetic variation in populations. A heterozygosity of 0.500–0.800 indicates that the genetic diversity of this population was high [20]. At the population level, from the Ho (0.760–0.845) and He (0.857–0.882) of these four wild populations, the genetic diversity of all the populations was at a high level. The heterozygosity values of the six farmed populations (Ho: 0.620–0.855; He: 0.642–0.842) were lower than those of the wild populations but also at a moderately high level. From the average heterozygosity of the four wild populations, He was higher than Ho, indicating a substantial heterozygote deficiency across the entire population. In order to supplement natural resources, large numbers of bighead carp juveniles are introduced into the Yangtze River every year, which may have had a negative impact on the genetics of natural populations. Moreover, both XZ and HG had a significantly lower He than Ho, which was consistent with the results of Zhu et al. [21] found the same Ho  <  He in the cultured populations from Shanggao (SG) and Suzhou (SZ) because of artificial selection and inbreeding, a small effective population, and null alleles. Although no genetic bottleneck event was detected recently, it was likely that long-term human intervention has resulted in reduced genetic diversity.

The study reported that the average numbers of alleles and expected heterozygosity per population in freshwater fishes were (9.1 ± 6.1) and (0.54 ± 0.25), respectively [22]. The mean values of allele number and expected heterozygosity of the four wild bighead carp populations in this study were 12.253 and 0.869, respectively, which were higher than those of other freshwater fishes in mainland China. It is interesting to note that the number of alleles and expected heterozygosity in this study were also at high levels in some of the aquaculture populations (HH, QC, CD). This may be due to the fact that in addition to the decisions made by the managers of the breeding farms, good conditions for eggs, larvae, and fry reduce the impact of selection on aquaculture populations that are subjected to natural conditions of environmental stresses, so that individuals that should be washed out of the population remain and then maintain a high level of genetic diversity in the population [23]. Another reason for the high genetic diversity of aquaculture populations may be the hybridization of populations that are sources of genetic differences [24].

In the WRIGHT classification criteria, a Fst between 0 and 0.05 indicates weak genetic differentiation between populations, 0.05–0.15 indicates moderate genetic differentiation, and 0.15–0.25 indicates high genetic differentiation. We found that the level of genetic differentiation in wild populations of bighead carp in the middle reaches of the Yangtze River was generally low (Fst < 0.05,), indicating extensive gene flow among the sampled populations. The Fst values all reached the significance level (*p* < 0.05). Our results were consistent with those found for bighead carp in the middle and lower reaches of the Yangtze River [21]. The genetic differentiation between the CH and HG populations exceeded the bottom line of moderate genetic differentiation, and there was great genetic differentiation. Meanwhile, CH and HG showed the same performance in phylogenetic tree, Bayesian clustering, and PCoA multiple analysis, and both of them showed unique taxa, which indicated that these two populations were genetically significantly differentiated. Before 2018, high-density and high-input cage culture was predominantly practiced in HG. This intensive farming method, coupled with the use of a limited breeding stock for bighead carp, led to the degradation of germplasm resources and a reduction in genetic diversity. CH is a shallow-water lake that has experienced changes in fish community structure due to prolonged enclosure and fence aquaculture practices, as well as environmental pollution. He et al. [25] found that the dominant fish species of Changhu Lake were mainly small fish or young individuals of large fish, and the fish community had been disturbed. These factors also contributed to the limited genetic diversity observed in the breeding stock of cultured bighead carp.

Despite the sharp decline in fish resources in recent decades, bighead carp in the middle Yangtze River still maintained a high genetic diversity, indicating that the fish has a high genetic capacity and adaptive potential. Compared with other fish species in the Yangtze River, such as grass carp and carp, bighead carp has been intensively farmed to a relatively low extent in recent years, and the effects of population recovery and germplasm resource conservation have been significant [26]. Although the level of genetic differentiation of wild populations of bighead carp in the middle reaches of the Yangtze River was generally low in this study (Fst < 0.05), the farmed population showed a reduced genetic diversity and genetic bottlenecks. In the future fisheries management and aquaculture management of bighead carp in the Yangtze River, it is necessary to strengthen the monitoring of the genetic diversity of farmed and wild populations and to expand the germplasm repository and genetic management unit. An integrated technical system for genetic diversity conservation, adaptive improvement, and ecological safety should be gradually established to promote the rapid development of aquaculture.

## 5. Conclusions

Eight polymorphic microsatellite markers were used to compare and analyze the genetic diversity and divergence of four wild and six farmed bighead carp populations in the middle reaches of the Yangtze River. Our data showed that the polymorphism levels of the cultured populations were generally lower than those of the wild populations. Bayesian clustering (K = 4), UPGMA phylogeny, and PCoA showed that the wild populations (ZX, DTH, SS) had distinct genetic clustering, while the cultured populations exhibited a mixed ancestry. Measures: Prioritize the conservation of wild populations to maintain the natural gene flow. Implement genetic monitoring and breeding strategies to improve the diversity of cultured fish. Promote sustainable aquaculture practices that integrate wild genetic resources to mitigate inbreeding risks. This study will provide an important reference benchmark for the conservation and sustainable utilization of bighead carp germplasm resources.

## Figures and Tables

**Table 1:** Sample information of ten populations of bighead carp in this study.

Population Number	Type of Populations	Population Abbreviations	Sample Location	Sample Size	Date
1	wild	ZX	Zhong District, Chongqing	32	2013
2		DTH	Northeastern Hunan Province’s Dongting Lake	32	2013
3		SS	Shishou City, Jingzhou	32	2019
4		WH	Five Lakes Fishery, Xiantao	32	2019
5	Cultured (market collection or adult)	HH	Honghu Lake	32	2019
6		XZ	Xinzhou Fish Farms, Wuhan	32	2019
7	Cultured (larvae)	CH	Changhu Fishery, Jingzhou	32	2019
8		QC	Herb County, Huanggang	32	2019
9		CD	Caidian District, Wuhan	32	2019
10		HG	Huanggang Fish Farms	32	2019

**Table 2:** Characterization of 8 microsatellites of bighead carp used in this study.

Locus	Repeat Motif	Primer Sequence (5′–3′)	Sequence Length (bp)	Annealing Temperature (°C)	Fluorochromes
Hysd6-1	(AGAAT)_12_	F:ATACAGCCATGACATGAACAC	191–266	50	HEX
		R:AACAAAGGAAATCTGCGTG			
Hysd293-1	(AAGAG)_70_	F:AACGAACTCATTTCCAGACCAG	115–220	49	FAM
		R:GCCAACATACATAAAGTACATCCC			
Hysd443-2	(AAGAG)_135_	F:TGACATTTATCCAAGTTTTA	190–260	49	FAM
		R:GATTTAGTTCTCAAGCATTT			
Hysd792-1	(GAAGA)_45_	F:ATAACTGAATCATTCCATCGCC	84–164	49	FAM
		R:AGCCTAACCTGCCCTTTACTTG			
Arsd12	(TTTC)_10_	F:CAGCCTAATGTTTCACAGTCTT	210–306	50	HEX
		R:CAGGGTTTGTGCCTAGATGT			
Arsd23	(TATC)_13_	F:TTTAGCCTATGTGAACGATGTG	201–254	48	FAM
		R:TGGGGAGAAACTTTAGCGAC			
Arsd26	(TCTT)_8_	F:CAAACTGTCAAACGACCACG	259–303	50	HEX
		R:GAACCTTTCACTTCTCACGATG			
Arsd241-1	(GATA)_12_	F:AACAAAAGTTTCAACGCAGGT	227–251	50	HEX
		R:GCCTCTCTGTGTCTGTGTAAGTG			

**Table 3:** Mean genetic parameters for 8 microsatellite markers of bighead carp.

Locus	Na	Ne	He	Ho	PIC	Fst
Hysd6	27	10	0.902	0.881	0.842	0.063
Hysd293-1	35	14.7	0.934	0.865	0.852	0.072
Arsd12	21	10	0.905	0.840	0.810	0.080
Arsd23	10	6.9	0.856	0.754	0.693	0.156
Arsd26	17	7.3	0.865	0.759	0.748	0.101
Hysd443-2	18	11.6	0.915	0.831	0.830	0.072
Arsd241	11	5.8	0.828	0.681	0.664	0.161
Hysd792-1	20	7.6	0.870	0.802	0.796	0.057
Average	19.9	9.2	0.884	0.802	0.779	0.095

**Table 4:** Genetic diversity of ten populations in bighead carp.

Type of Populations	Population Abbreviations	Sample Size	Na	Ne	He	Ho	PIC
Wild	ZX	32	11.88	7.23	0.871	0.822	0.842
	DTH	32	12.75	8.06	0.882	0.827	0.853
	SS	32	12	7.31	0.865	0.760	0.834
	WH	32	12.38	6.83	0.857	0.845	0.827
	Average	32	12.253	7.358	0.869	0.814	0.839
Cultured	HH	32	9.5	5.54	0.825	0.793	0.790
	XZ	32	8.88	4.80	0.798	0.832	0.756
	CH	32	8.5	4.84	0.642	0.620	0.613
	QC	32	10.38	6.12	0.833	0.828	0.798
	CD	32	9.43	6.35	0.842	0.836	0.807
	HG	32	6.38	3.78	0.731	0.855	0.674
	Average	32	8.845	5.238	0.779	0.794	0.740

**Table 5:** Nei’s genetic distance (below diagonal) and pairwise Fst values (above diagonal) of ten populations in bighead carp.

Population	HH	XZ	CH	QC	CD	ZX	DTH	SS	WH	HG
Abbreviations										
HH		0.077 *	0.228 *	0.035 *	0.053 *	0.034 *	0.037 *	0.036 *	0.028 *	0.097 *
XZ	0.492		0.231 *	0.074 *	0.073 *	0.067 *	0.056 *	0.064 *	0.070 *	0.171 *
CH	0.791	0.778		0.202 *	0.212 *	0.196 *	0.171 *	0.185 *	0.187 *	0.293 *
QC	0.265	0.484	0.782		0.038 *	0.029 *	0.020 *	0.024 *	0.038 *	0.128 *
CD	0.399	0.495	0.789	0.306		0.026 *	0.021 *	0.019 *	0.05 *	0.108 *
ZX	0.284	0.477	0.783	0.263	0.253		0.011 *	0.008 *	0.032 *	0.107 *
DTH	0.315	0.409	0.768	0.207	0.224	0.18		0.003 *	0.021 *	0.109 *
SS	0.295	0.452	0.774	0.23	0.201	0.146	0.122		0.025 *	0.108 *
WH	0.239	0.495	0.776	0.321	0.431	0.323	0.251	0.261		0.119 *
HG	0.498	0.77	0.827	0.774	0.609	0.639	0.699	0.647	0.748	

* Represents Fst values were significantly different from zero (*p* < 0.05).

**Table 6:** Hardy–Weinberg equilibrium test of ten populations in bighead carp.

Population	Parameter	Hysd6	Hysd293-1	Arsd12	Arsd23	Arsd26	Hysd443-2	Arsd241	Hysd792-1
HH	Na	10	13	9	7	9	12	6	10
	Ne	6.850	6.263	6.919	4.080	4.541	6.827	4.231	4.582
	He	0.878	0.854	0.869	0.767	0.792	0.867	0.776	0.794
	Ho	0.938	0.969 *	0.938	0.781 *	0.688 **	0.781 **	0.531 **	0.719
	PIC	0.850	0.823	0.839	0.719	0.754	0.839	0.736	0.759
XZ	Na	11	11	9	7	10	9	6	8
	Ne	5.404	6.759	5.971	5.007	4.267	3.828	3.309	3.835
	He	0.868	0.866	0.846	0.813	0.778	0.751	0.709	0.751
	Ho	1 **	0.969 **	1.000	0.844 **	0.719 **	0.688 **	0.719 *	0.719 **
	PIC	0.838	0.835	0.811	0.772	0.733	0.710	0.657	0.698
CH	Na	11	13	8	2	5	14	2	13
	Ne	6.261	7.119	4.979	1.174	2.566	9.062	1.064	6.461
	He	0.854	0.874	0.812	0.151	0.620	0.904	0.062	0.859
	Ho	0.936	0.742 **	0.742	0.161	0.656	0.875 *	0 *	0.844
	PIC	0.821	0.845	0.771	0.137	0.562	0.880	0.059	0.829
QC	Na	16	10	8	7	11	12	7	12
	Ne	7.670	4.180	4.491	5.842	6.850	7.969	3.568	8.393
	He	0.883	0.773	0.788	0.842	0.868	0.888	0.732	0.888
	Ho	0.875	0.719	0.813	0.903	0.969	0.875	0.563 *	0.903
	PIC	0.857	0.736	0.745	0.806	0.838	0.862	0.679	0.862
CD	Na	11	11	10	6		12	6	10
	Ne	8.641	8.063	6.628	3.984	6.827	7.062	3.501	6.113
	He	0.909	0.890	0.863	0.761	0.867	0.872	0.726	0.850
	Ho	0.938	0.844 *	0.813	0.906	0.844	0.688 **	0.750	0.906 *
	PIC	0.886	0.864	0.832	0.707	0.836	0.843	0.667	0.819
ZX	Na	13	18	12	8	12	12	9	11
	Ne	6.282	11.838	7.066	6.322	6.006	9.615	5.309	5.361
	He	0.903	0.930	0.873	0.856	0.847	0.910	0.825	0.826
	Ho	0.719 **	0.875 *	0.742 **	0.839	0.813	0.781 *	0.968	0.844
	PIC	0.879	0.910	0.844	0.822	0.814	0.887	0.789	0.790
DTH	Na	14	20	13	7	11	16	8	13
	Ne	7.938	13.213	9.309	6.077	6.889	9.468	5.069	6.502
	He	0.907	0.939	0.907	0.849	0.869	0.909	0.816	0.860
	Ho	0.813	0.938	0.875	0.719 **	0.871	0.839	0.875	0.688
	PIC	0.883	0.919	0.883	0.814	0.838	0.885	0.776	0.828
SS	Na	14	21	13	9	10	12	7	10
	Ne	7.642	10.952	8.258	6.919	4.571	9.894	4.631	5.620
	He	0.899	0.923	0.893	0.869	0.794	0.913	0.797	0.836
	Ho	0.688	0.813	0.750	0.781	0.656 **	0.938	0.710	0.742
	PIC	0.874	0.902	0.867	0.839	0.752	0.890	0.751	0.801
WH	Na	14	16	14	7	13	13	9	13
	Ne	7.94	8.90	7.64	4.30	4.90	8.87	6.01	6.08
	He	0.89	0.90	0.88	0.78	0.81	0.90	0.85	0.85
	Ho	0.97	0.91	0.844 *	0.906 **	0.75	0.91	0.719 **	0.75 **
	PIC	0.86	0.88	0.86	0.73	0.77	0.88	0.81	0.82
HG	Na	6	14	5	5	4	5	6	6
	Ne	3.562	5.769	3.419	2.731	2.764	3.246	3.988	4.746
	He	0.731	0.840	0.719	0.644	0.648	0.703	0.762	0.802
	Ho	0.906 **	0.875	0.875 *	0.688 **	0.625 *	0.938 **	1 **	0.936 **
	PIC	0.672	0.806	0.655	0.578	0.583	0.630	0.710	0.758

* Represents deviating from Hardy–Weinberg equilibrium at the level of *p* < 0.05. ** Represents deviating from Hardy–Weinberg equilibrium at the level of *p* < 0.01.

**Table 7:** Analysis of mutation–drift equilibrium of ten populations in bighead carp.

Population Abbreviations	Sign Test						Wilcoxon Test		
	IAM		TPM		SMM		IAM	TPM	SMM
	He/Hd	*p*	He/Hd	*p*	He/Hd	*p*	*p*	*p*	*p*
HH	8/0	0.018 *	3/5	0.574	3/5	0.188	0.004 **	0.250	0.313
XZ	8/0	0.017 *	4/4	0.421	2/6	0.055	0.004 **	0.641	0.980
CH	6/2	0.212	5/3	0.486	2/6	0.065	0.055	0.844	0.020 *
QC	8/0	0.017 *	6/2	0.288	3/5	0.194	0.004 **	0.250	0.461
CD	8/0	0.018 *	8/0	0.016 *	5/3	0.572	0.004 **	0.004 **	0.195
ZX	8/0	0.017 *	5/3	0.603	3/5	0.189	0.004 **	0.055	0.313
DTH	8/0	0.020 *	7/1	0.105	4/4	0.431	0.004 **	0.011 *	1.000
SS	8/0	0.019 *	6/2	0.308	3/5	0.192	0.004 **	0.074	0.770
WH	7/1	0.113	6/2	0.315	2/6	0.058	0.012 *	0.547	0.039 *
HG	7/1	0.097	7/1	0.104	5/3	0.568	0.008 **	0.195	0.547

Note: *H_e_*/*H_d_*: The ratio of number of loci with excess heterozygosity to number of loci with heterozygosity deficiency. * represents deviating from mutation–drift equilibrium at the level of *p* < 0.05. ** represents deviating from mutation–drift equilibrium at the level of *p* < 0.01.

## Data Availability

The raw data supporting the conclusions of this article will be made available by the authors on request.
